# Case report: Rare Guillain-Barré syndrome variants and mild encephalitis/encephalopathy with a reversible splenial lesion as the para-infectious manifestations of SARS-CoV-2 infection

**DOI:** 10.3389/fimmu.2024.1458231

**Published:** 2024-10-04

**Authors:** Wei Si, Yuanrui Li, Ganqin Du

**Affiliations:** ^1^ Department of Neurology, The First Affiliated Hospital, College of Clinical Medicine of Henan University of Science and Technology, Luoyang, Henan, China; ^2^ Department of Neurology, Guizhou Provincial People’s Hospital, Guiyang, Guizhou, China

**Keywords:** SARS-CoV-2, Guillain-Barré syndrome, polyneuritis cranialis, acute panautonomic neuropathy, case report

## Abstract

**Background:**

The Coronavirus disease 19 (COVID-19), caused by the severe acute respiratory syndrome coronavirus 2 (SARS-CoV-2), is a threat to human health. Although the COVID-19 pandemic is finished, some peoples are still suffering from this disease. Herein, we report the first case of SARS-CoV-2-associated Guillain-Barré syndrome (GBS) presenting as polyneuritis cranialis (PNC) and acute panautonomic neuropathy (APN) variants, accompanied by mild encephalitis/encephalopathy with a reversible splenial lesion (MERS) and hyponatremia.

**Case presentation:**

A 32-year-old female patient with symptoms indicating multiple cranial nerve involvement, as well as sympathetic and parasympathetic nervous system dysfunction, was diagnosed as SARS-CoV-2-associated GBS presenting as PNC and APN variants, accompanied by MERS and hyponatremia. Following treatment with immunoglobulin, methylprednisolone, and symptomatic care, the patient’s inflammatory cytokines and serum sodium became normal. However, some residual symptoms such as postural hypotension, fatigue, and mild dysarthria persisted at the 9-month follow-up.

**Conclusion:**

This case highlights the unique presentation of SARS-CoV-2 infection. The involvement of both the central nervous system (CNS) and the peripheral nervous system (PNS) in this case underscores the complex neurological manifestations of COVID-19. Although the exact underlying pathogenesis of this case is unclear, inflammatory cytokines, particularly IL-6, may be implicated. Further research is needed to better understand the mechanisms underlying these complications and to optimize treatment strategies for affected patients.

## 1 Introduction

The Coronavirus disease 19 (COVID-19), caused by the severe acute respiratory syndrome coronavirus 2 (SARS-CoV-2), is a global threat to human health. Although the pandemic is finished, some peoples are still suffering from this disease. As of April 13, 2024, 704,753,890 confirmed cases and 7,010,681 deaths have been reported worldwide (https://www.worldometers.info/coronavirus/).

Guillain-Barré syndrome (GBS) is a rare but potentially life-threatening group of immune-mediated polyneuropathies. Based on the clinical and electrophysiological characteristics, GBS can be classified into several subtypes including acute inflammatory demyelinating polyradiculoneuropathy (AIDP), acute motor axonal neuropathy (AMAN), acute motor sensory axonal neuropathy (AMSAN), Miller Fisher Syndrome (MFS), and other rare variants like acute panautonomic neuropathy (APN), polyneuritis cranialis (PNC), Bickerstaff brainstem encephalitis (BBE), and pharyngeal-cervical-brachial GBS. Most cases of GBS occur subsequently to a previous respiratory or gastrointestinal tract infection. Starting from the first case of GBS following SARS-CoV-2 infection, an increasing number of SARS-CoV-2-associated GBS have been published, and AIDP appears to be most common (1). However, rare variants of GBS, such as PNC (2, 3) and APN have rarely or not been reported in patients with SARS-CoV-2 infection. Herein, we report the first case of SARS-CoV-2-associated GBS presenting as APN and PNC variants, accompanied by mild encephalitis/encephalopathy with a reversible splenial lesion (MERS) and hyponatremia.

## 2 Case description

**Figure 1:**
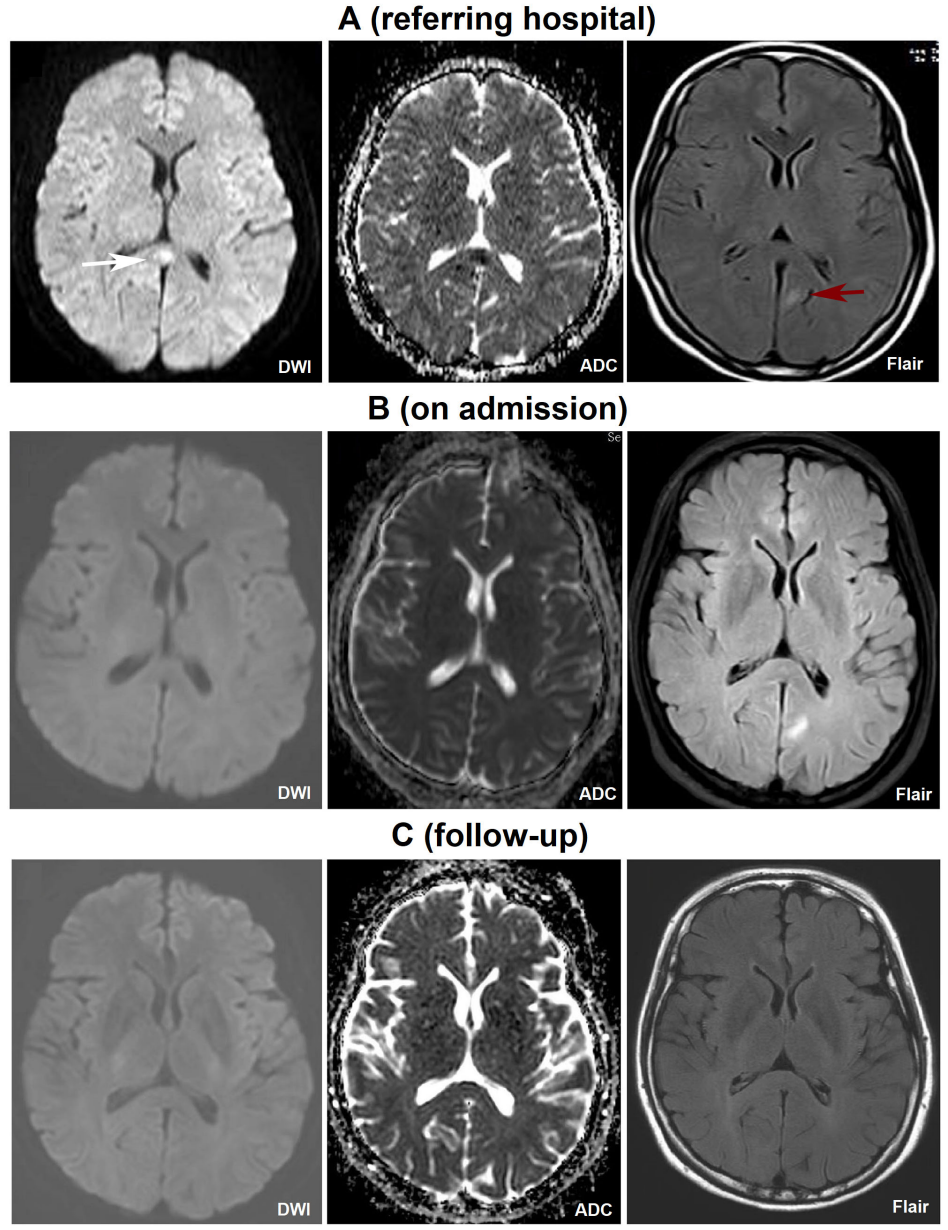
Brain MRI images at different time-points. Images taken from the referring hospital **(A)**, on admission **(B)** and 3-month follow-up **(C)**. The MRI signals showing cytotoxic edema in the SCC (white arrow), characterized by hyperintensity in the DWI sequence and hypointensity in the ADC sequence, resolved rapidly. In contrast, the signal indicating acute demyelination in the SCC and left occipital lobe (red arrow), identified by hyperintensity in the Flair sequence, persisted for a longer period. MRI, magnetic resonance imaging; SCC, the splenium of the corpus callosum.

A 32-year-old female patient presented to our hospital due to “runny nose and sore throat for 8 days, and confusion for 2 days” at June of 2023. Eight days before admission, she had experienced a runny nose and sore throat, with a normal temperature of 37.2°C. She tested positive for SARS-CoV-2 antigen and also reported symptoms such as poor appetite, fatigue, dizziness, night sweats, and 2-3 episodes of paroxysmal syncope, without limb convulsions or urinary/fecal incontinence. Two days before admission, the patient was found confused, sitting on the floor with urinary incontinence. She was taken to another hospital, where her supine blood pressure (BP) was 80/40 mmHg, heart rate was 92 beats/min, SPO2 was 95%, and serum sodium was 116mmol/L. Additionally, her brain magnetic resonance imaging (MRI) showed abnormal signals in the splenium of the corpus callosum (SCC) and the left occipital lobe (**Figure 1A**). Despite receiving symptomatic treatments such as BP elevation and sodium supplementation, her BP and serum sodium levels remained low. She also developed delirium, along with symptoms of coughing while drinking, dysphagia, urinary retention, and diarrhea, leading to her transfer to our hospital.

Upon admission, the patient admitted that she had received three doses of vero cell inactivated vaccine SARS-CoV-2 vaccine (Sinovac, China) at 25/24/18 months ago and had a history of mild SARS-CoV-2 infection 6 months earlier. Her supine BP was 78/40 mmHg. Physical examination revealed dry skin with absence of sweating, increased bowel sounds, delirium, poor coordination during the advanced cortical function examination, dysarthria, pupillary alterations (left pupil diameter was 5.5 mm with absent pupillary light response, right pupil was 4.0 mm with reduced pupillary light response), incomplete eyelid closure (bilaterally), symmetric frontal striae and nasolabial fold getting shallow (bilaterally), unable to blow up the cheeks and show the teeth, weakened pharyngeal reflex, incomplete tongue extension, decreased muscle strength (grade IV) and tone in all limbs, absent abdominal and bilateral tendon reflexes. Superficial sense impairment was noticed in the ends of limbs but her deep sense was normal. She was unable to finish bilateral finger-nose test, heel-knee-tibia test, and Romberg test. Her plantar reflex (both sides) and meningeal irritation sign were negative, skin scratch sign and nasopharyngeal SARS-CoV-2 polymerase chain reaction test were positive.

Laboratory tests revealed the following abnormalities: white blood cells, 12.37×10^9^; neutrophils, 11.46×10^9^; lymphocytes, 0.59×10^9^; serum sodium, 116mmol/L; high-sensitivity C-reactive protein (Hs-CRP), 37.55 mg/L; erythrocyte sedimentation rate (ESR), 46 mm/h, IL-6, 15.28 pg/mL; T3, 1.65 pg/mL; thyroid stimulating hormone, 0.15 uIU/mL. Her anti-Ro52 IgG (>5000 AU/mL) and anti-SSA/Ro60 IgG (>200 AU/mL) were positive. Cerebrospinal fluid (CSF) analysis (routine/biochemical test, staining, culture, and next-generation sequencing) didn’t show albuminocytologic dissociation (protein concentration: 485 mg/L; normal range: 120-600 mg/L) or any other abnormities. Additionally, other tests such as liver/renal/coagulation functions, tumor markers, respiratory examination, abdominal and pelvic/cervical vascular ultrasounds, and electrocardiogram, were normal. Serum and CSF autoimmune encephalitis antibodies and serum anti-neutrophil cytoplasmic antibodies were negative. No epileptiform discharge was observed. Two day after admission, brain MRI showed high signals in the SCC and the left occipital lobe (**Figure 1B**). The nerve conduction study (NCS) at the 8th day of hospitalization revealed some abnormalities. In the motor NCS, decreased conduction velocity was observed in the median nerves, tibial nerves, and the right side common peroneal nerve. In the sensory NCS, decreased amplitude was noted in the median, ulnar, and tibial nerves, along with decreased conduction velocity in both the superficial peroneal and tibial nerves on both sides (**Table 1**).

**Table 1:** NCS at the 8th day of hospitalization.

NCS	Nerve	Stimulation site	Recording site	Amplitude*			Latency (ms)			Conduction velocity (m/s)			F-wave latency (ms)		F-wave persistence (%)	
				L	R	Normal	L	R	Normal	L	R	Normal	L	R	L	R
Motor NCS	Median	Wrist	APB	9.2	7.1	> 5.0	2.92	2.71	< 4.0	—	—	—	27.1	25.2	80	80
		Elbow	Wrist	9.5	7.1	—	6.54	6.75	—	48.0	49.2	> 48.0	—	—	—	—
	Ulnar	Wrist	ADM	7.5	7.2	> 7.0	2.62	1.95	< 3.0	—	—	—	—	—	—	—
		AF	Wrist	9.2	8.0	—	7.38	5.83	—	47.8	43.5	> 48.0	—	—	—	—
	Common peroneal	Ankle	EDB	3.3	3.7	> 3.0	3.23	3.41	< 5.0	—	—	—	—	—	—	—
		Below-knee	Ankle	3.0	3.8	—	9.92	9.35	—	44.5	41.8	> 44.0	—	—	—	—
	Tibial	Ankle	AHB	8.9	13.8	> 6.0	3.48	3.05	< 6.0	—	—	—	46.5	46.8	100	100
		PF	Ankle	6.8	11.2	—	11.2	11.3	—	40.7	38.5	> 42.0	—	—	—	—
Sensory NCS	Median	Second finger	Wrist	7.6	8.8	> 16.0	2.27	2.02	—	50.2	46.8	> 45.0	—	—	—	—
	Ulnar	Fifth finger	Wrist	4.5	4.0	> 7.0	1.73	1.63	—	47.7	45.2	> 44.2	—	—	—	—
	Superficial peroneal	Ankle	Dorsum pedis	2.3	2.2	> 1.2	1.51	2.48	—	35.1	32.1	> 45.4	—	—	—	—
	Tibial	First toe	Ankle	0.6	0.6	> 0.8	2.53	2.3	—	30.4	33.5	> 35.1	—	—	—	—

* Motor NCS = mV, Sensory NCS = μV.

Red color highlights for abnormal values.

NCS, nerve conduction study; L, lest side; R, right side; APB, abductor pollicis brevis; ADM, abductor digiti minimi; AF, antecubital fossa; EDB, extensor digitorum brevis; AHB, abductor hallucis brevis; PF, Popliteal fossa.

Her muscle strength and tone in all limbs rapidly returned to a state close to normal. Based on all clinical symptoms and examination results, SARS-CoV-2-associated GBS presenting as PNC and APN variants, accompanied by para-infectious encephalopathy, was considered. After receiving immunoglobulin (0.4g/kg/day IV for 5 days), methylprednisolone (500mg/day IV for 3 days) and symptomatic treatments (such as norepinephrine infusion and sodium supplement), the patient regained her consciousness and her cranial nerve damages were slightly improved. Her serum sodium became normal. No urinary retention was presented. Immunoglobulin therapy (0.4g/kg/day IV for 5 days) was continued, and droxidopar, acupuncture, pregabalin, mecobalamin and vitamin B1 were added. The BP was maintained at about 100/70 mmHg in the supine position and 70/50 mmHg in the sitting position. Re-examination at discharge showed the laboratory indicators such as serum sodium, ESR, Hs-CRP, IL-6 were at normal range.

Three months after discharge, brain MRI showed normal signals in the SCC and the left occipital lobe (**Figure 1C**). The NCS results revealed improvements as compared with that observed at hospitalization (**Table 2**). Nine months after discharge, some abnormalities such as postural hypotension, fatigue, mild dysarthria, abnormal pupil diameter (left, 3 mm; right, 2.5 mm), and superficial sense impairment at the ends of limbs, were still observed; While, other physical examination results were almost normal. Re-examination of the antinuclear antibodies showed her anti-Ro52 IgG reduced to 48.9 AU/ml and anti-SSA/Ro60 IgG to 31.5 AU/ml.

**Table 2:** NCS at follow-up.

NCS	Nerve	Stimulation site	Recording site	Amplitude*			Latency (ms)			Conduction velocity (m/s)			F-wave latency (ms)		F-wave persistence (%)	
				L	R	Normal	L	R	Normal	L	R	Normal	L	R	L	R
Motor NCS	Median	Wrist	APB	12.0	10.1	> 5.0	3.86	3.81	< 4.0	—	—	—	28.1	30.4	100	100
		Elbow	Wrist	11.0	9.5	—	8.17	8.08	—	55.2	56.9	> 48.0	—	—	—	—
	Ulnar	Wrist	ADM	8.5	8.5	> 7.0	3.01	2.65	< 3.0	—	—	—	—	—	—	—
		AF	Wrist	9.9	9.1	—	8.12	7.94	—	50.4	59.3	> 48.0	—	—	—	—
	Common peroneal	Ankle	EDB	4.3	4.8	> 3.0	3.78	3.6	< 5.0	—	—	—	—	—	—	—
		Below-knee	Ankle	5.4	5.0	—	10.3	9.38	—	44.8	50.5	> 44.0	—	—	—	—
	Tibial	Ankle	AHB	16.3	17.6	> 6.0	4.19	3.77	< 6.0	—	—	—	47.9	50.5	100	100
		PF	Ankle	14.1	15.4	—	12.3	12.6	—	45.3	42.4	> 42.0	—	—	—	—
Sensory NCS	Median	Second finger	Wrist	12.5	14.8	> 16.0	2.49	2.78	—	55.1	56.9	> 45.0	—	—	—	—
	Ulnar	Fifth finger	Wrist	6.2	5.5	> 7.0	2.52	2.34	—	61.3	67.1	> 44.2	—	—	—	—
	Superficial peroneal	Ankle	DP	3.1	3.3	> 1.2	2.85	3.43	—	66.2	43.3	> 45.4	—	—	—	—
	Tibial	First toe	Ankle	1.2	1.1	> 0.8	2.11	2.53	—	40.2	40.6	> 35.1	—	—	—	—

* Motor NCS = mV, Sensory NCS = μV.

Red color highlights abnormal values.

NCS, nerve conduction study; L, lest side; R, right side; APB, abductor pollicis brevis; ADM, abductor digiti minimi; AF, antecubital fossa; EDB, extensor digitorum brevis; AHB, abductor hallucis brevis; PF, Popliteal fossa; DP, Dorsum pedis.

## 3 Discussion

SARS-CoV-2 can trigger GBS in either post-infectious or para-infectious pattern, but the underlying pathogenesis has not been fully understood. Molecular mimicry, cytokine storm, blood‐nerve barrier disruption, direct neurotropism and neurovirulence of SARS-CoV-2, microvascular dysfunction, and autoantibody production may contribute to the occurrence of GBS following SARS-CoV-2 infection (4). PNC is an unusual variant of GBS that primarily affects the motor cranial nerves, such as the facial nerve, glossopharyngeal nerve, and vagus nerve. APN is another rare variant of GBS that affects both the sympathetic and parasympathetic nervous systems. Postural hypotension is the most common symptom of APN. Up to now, only a few cases of GBS presenting as PNC variant has been reported in SARS-CoV-2 infection patients (2, 3). No APN variant has been reported as associated with SARS-CoV-2 infection. In this case, the patient presented to our hospital with symptoms indicating multiple cranial nerve involvement (such as dysarthria, pupillary alterations, incomplete eyelid closure, and symmetric frontal striae and nasolabial fold getting shallow), as well as sympathetic and parasympathetic nervous system dysfunction (such as dry skin with absence of sweating, increased bowel sounds, and postural hypotension). Despite the presence of limb weakness, which is not typically seen in APN and PNC variants of GBS, and the absence of albuminocytologic dissociation in the CSF analysis, a diagnosis of para-infectious SARS-CoV-2-associated GBS presenting as PNC and APN variants was made based on the patient’s medical history, physical examination and neurophysiologic findings. The patient’s limb weakness upon admission was presumed to be related to factors such as delirium, hypotension, hypokalemia, and hyponatremia at that time. This presumption was supported by the patient’s lack of complaints regarding limb weakness prior to admission, the improvement in muscle strength and tone in all four limbs before immunotherapy, and the electromyography findings showing no significant motor or sensory involvement. The patient’s impaired consciousness and clinical presentation also needed to be differentiated from BBE, but the absence of pyramidal signs throughout the disease ruled out BBE as a possibility.

While SARS-CoV-2 mainly affects the respiratory system, it also leads to extensive extrapulmonary injuries affecting both the CNS and the PNS. However, cases of SARS-CoV-2-associated GBS with CNS involvement are rarely reported. Paterson et al. documented a 52-year-old male SARS-CoV-2 positive patient, with combined GBS and acute disseminated encephalomyelitis (ADEM)-like illness (5). In another study, Pimentel et al. systematically reviewed 436 patients with GBS associated with COVID-19, and they found concomitant CNS involvement in 35 cases (6). Manifestations such as headache, dizziness, delirium, impaired or loss of consciousness, seizures, and acute cerebrovascular disease are common but nonspecific indicators of CNS involvement (7).

MERS is a clinicoradiological syndrome with diverse etiology. Since the COVID-19 pandemic, many cases of MERS following SARS-CoV-2 infection have been reported (8–11). In this case, the patient’s transcutaneous oxygen saturation and arterial blood gas analysis were almost normal. Additionally, her CSF examinations and autoimmune encephalitis antibodies were negative. She developed encephalopathic manifestations such as blurred consciousness and delirium at 6 days post-SARS-CoV-2 infection. Furthermore, the abnormal signals in the SCC and the left occipital lobe, as shown by brain MRI, disappeared quickly. Based on these findings, a diagnosis of SARS-CoV-2-associated MERS was made. Recently, Hayashi et al. reported a case of MERS in a patient with hypoglycemia who died of sepsis. Based on imaging scans and postmortem pathology results, authors declared transient demyelination following cytotoxic edema in the SCC is a pathogenesis of MERS associated with hypoglycemia (12). In our case, the patient’s MRI signals indicating the presence of cytotoxic edema in the SCC, that is hyperintensity in the DWI sequence and hypointensity in the ADC sequence, disappeared quickly. Whereas the signal suggesting acute demyelination in the SCC and left occipital lobe, as indicated by hyperintensity in the Flair sequence, persisted for a longer period. Although we can’t declare this as a rare case of combined central and peripheral demyelination following SARS-CoV-2 infection due to lack of histological results to support the diagnosis of CNS demyelination, the extensive involvement of both the CNS and the PNS makes this case particularly unique.

Hyponatremia has been previously reported in SARS-CoV-2-associated MERS patients (8). In our case, the patient’s hyponatremia persisted despite several rounds of slowly correction of serum sodium. Therefore, central hyponatremia was considered. Although it is not possible to establish a direct association between SARS-COV-2 infection, MERS and hyponatremia in this patient, we believe that inflammatory cytokines, particularly IL-6, may be implicated for the following reasons: Firstly, numerous inflammatory cytokines are released during infection, which can lead to a cytokine storm and play a crucial role in the pathogenesis of SARS-CoV-2 infection (13). Secondly, inflammatory cytokines-related cellular swelling and cytotoxic edema have been proposed as the underlying pathophysiology of MERS (14). Thirdly, elevated expression of IL-6, acting as a second messenger to trigger the release of vasopressin, has been hypothesized to be involved in the development of hyponatremia (15).

## 4 Conclusions

This case highlights the unique presentation of SARS-CoV-2 infection. The involvement of both the CNS and the PNS in this case underscores the complex neurological manifestations of COVID-19. Further research is needed to better understand the mechanisms underlying these complications and to optimize treatment strategies for affected patients.

## Data Availability

The original contributions presented in the study are included in the article/Supplementary Material. Further inquiries can be directed to the corresponding author.
